# Dermatological, Trichological and Quality of Life Consequences of GLP‐1 Receptor Agonist‐Mediated Weight Loss: A US Cross‐Sectional Survey

**DOI:** 10.1111/jocd.71145

**Published:** 2026-08-20

**Authors:** Janet Wangari Olivero, Jyotsna Paturi, Magali Moreau, Kun Qian, Stacy‐Ann Stevenson, Yaxian Zhen, Qian Zheng

**Affiliations:** ^1^ L'oréal Research & Innovation USA Clark New Jersey USA

**Keywords:** cutaneous changes, dermatological care, GLP‐1 receptor agonists, hair loss, medication‐driven weight loss, telogen effluvium, trichological changes

## Abstract

**Background:**

Glucagon‐like peptide‐1 receptor agonists (GLP‐1 RAs), including dual glucagon‐like peptide‐1/glucose‐dependent insulinotropic polypeptide (GLP‐1/GIP) receptor agonists, have revolutionized obesity management, resulting in significant medication‐driven weight loss (MDWL). Despite widespread reporting, the dermatological consequences of MDWL, including skin laxity and hair loss, remain poorly characterized in real‐world populations.

**Aims:**

To quantify the prevalence, onset, and phenotypic characteristics of skin and hair changes during MDWL with GLP‐1 and dual GLP‐1/GIP receptor agonists, and to evaluate patient‐reported treatment satisfaction and health‐related quality of life.

**Methods:**

A single‐center, cross‐sectional survey was administered between June 20 and July 15, 2025. A total of 504 adults who were using GLP‐1 RA or dual GLP‐1/GIP RA medications completed a 55‐item HIPAA‐compliant electronic questionnaire capturing demographics, medication history, weight loss, cutaneous and/or trichological changes, treatment satisfaction, and psychological well‐being.

**Results:**

The cohort was predominantly female (89.9%), with a mean age of 52.8 ± 11.2 years and a mean pre‐treatment BMI of 37.5 ± 8.1. Skin changes were reported by 38.7% of respondents, characterized primarily by facial volume loss (10.9%), sagging (8.3%), and jowling (7.7%). Onset occurred within the first 5 months of therapy for 55.1% of these participants. Skin changes correlated with age > 55 years and weight loss of > 20 lb/9.1 kg, with consistent prevalence across drug formulations. Hair changes were reported by 38.9% of the cohort, dominated by shedding (20.8%) and thinning (19.0%). Of those reporting shedding, 42.0% experienced onset despite being on treatment for only 1–3 months. Distinct racial phenotypes emerged in hair changes: White users frequently reported classic telogen effluvium‐type changes (shedding: 23.8%; thinning: 22.6%), whereas Black/African American users reported disproportionately higher rates of structural hair changes (dryness: 15.3%; breakage: 12.1%). Overall treatment satisfaction was exceptionally high at 92.0%, accompanied by significant improvements in body image (84.0%) and self‐confidence (68.5%).

**Conclusions:**

Dermatological cutaneous and trichological changes are frequent, early‐onset consequences of MDWL that correlate with weight loss speed and magnitude, exhibiting distinct phenotypes across age and race/ethnicity. These findings highlight the need for personalized proactive integrative dermatological care models that should be initiated early and maintained through the weight loss medication journey.

## 1 Introduction

The dermatological relationship with GLP‐1 receptor modulation is complex and bidirectional. On one hand, GLP‐1 RAs exhibit systemic anti‐inflammatory and immunomodulatory properties. Clinical trials and observational studies have demonstrated substantial therapeutic benefits in inflammatory dermatoses, including psoriasis (mediated via the inhibition of the IL‐17/IL‐23 axis and NF‐κB signaling) [1, 2, 3], hidradenitis suppurativa [4, 5], atopic dermatitis [6, 7] and acanthosis nigricans [8]. On the other hand, rapid, substantial weight loss induces distinct physiological changes in skin and hair [9, 10, 11]. Reports from post‐marketing surveillance and clinical cases highlight aesthetic and functional concerns, especially midfacial volume loss [12], increased skin laxity, and acute hair shedding resembling telogen effluvium (TE) [7, 13, 14, 15]. These structural changes are further compounded by potential nutritional deficiencies resulting from drug‐induced appetite suppression and subsequent severe caloric restriction [7, 14].

Despite this awareness, real‐world epidemiological data systematically characterizing the prevalence, temporal onset, and patient perceptions of these dermatological changes, particularly across diverse populations, remain limited. Such robust quantitative data can help drive future innovation in the identification of ingredients, formulations, or combinational regimens that can expand the MDWL patient toolbox.

To address this knowledge gap, we conducted a comprehensive cross‐sectional survey of 504 GLP‐1 RA and dual GLP‐1/GIP RA users. Our objective was to quantify patient‐reported cutaneous and trichological changes, define their trajectories over time, assess variations across race/ethnicity, and evaluate overall treatment satisfaction and quality‐of‐life outcomes.

## 2 Methods

### 2.1 Study Design

This single‐center, cross‐sectional survey study was administered electronically via a secure, electronic patient platform between June 20 and July 15, 2025. To ensure geographic and demographic diversity, a multichannel digital recruitment campaign was deployed. A total of 1951 outreach invitations yielded 885 pre‐screened patient interactions. A total of 504 participants successfully met all eligibility criteria and completed the survey, representing a 56.9% completion rate among pre‐screened individuals and a 25.8% overall response rate. Geographic analysis of participant zip codes demonstrated representation across 14 US states, with the majority of the cohort concentrated in Texas (70.8%), Arizona (12.3%), and New Jersey (11.3%), alongside smaller representations from New York, Tennessee, Florida, South Carolina, Massachusetts, Idaho, Vermont, Ohio, Oklahoma, North Carolina, and West Virginia.

Eligibility criteria required participants to be over the age of 18 years, currently using a prescribed weight loss medication (GLP‐1 RA or dual GLP‐1/GIP RA) for a minimum of 1 month, capable of completing the survey in English, and willing to provide verifiable proof of current prescription (e.g., prescription label, pharmacy receipt, or packaging matching legal name).

Consistent with applicable research ethics regulations for minimal‐risk surveys, this study was exempt from institutional review board oversight. Written informed consent was obtained from all participants prior to enrollment. Following enrollment, participants received a secure link to a 55‐item Sponsor‐provided self‐assessment questionnaire administered via Datacapt electronic survey software, compliant with both HIPAA and FDA 21 CFR Part 11 standards, to be completed within one week of enrollment. The questionnaire captured data on demographics, general health self‐rating, treatment variables, medication history, weight before and during treatment, skin quality and reactions, hair and scalp health, psychological well‐being, lifestyle behaviors, socioeconomic status, and treatment satisfaction. Questions included both closed‐ended items and open‐ended fields for qualitative elaboration. To ensure adequate statistical power for subgroup evaluations, a target enrollment of at least 500 completed responses was established. Participant data were de‐identified and analyzed in aggregate form. This study is reported in accordance with Strengthening the Reporting of Observational Studies in Epidemiology (STROBE) guidelines.

### 2.2 Statistical Analysis

Data analysis was conducted on the per‐protocol (PP) population (*n* = 504), defined as all participants who completed the study in accordance with the protocol. Categorical variables are reported as frequencies and percentages; continuous variables (e.g., body weight, weight loss magnitude) are reported as means with standard deviation (SD). The prevalence of skin changes was further examined by age group, weight loss magnitude, and medication type. Differences in hair change patterns across racial and ethnic subgroups were assessed using Pearson's Chi‐Square tests. Statistical significance was defined as *p* < 0.05. Treatment satisfaction and psychological well‐being outcomes were summarized descriptively across the full cohort.

## 3 Results

The demographic characteristics of the per‐protocol respondents (*n* = 504) are presented in Table 1. The participants were predominantly female (89.9%; *n* = 453), with a mean age of 52.8 ± 11.2 years. The largest age group was 55 to 64 years (29.8%; *n* = 150), followed by 45 to 54 years (28.6%; *n* = 144). The participants' self‐reported race and ethnicity were 67.7% White (*n* = 341), 24.6% Black or African American (*n* = 124), 4.8% Asian (*n* = 24), 2.6% multiracial (*n* = 13), and 0.2% each American Indian or Alaska Native and Other (*n* = 1 each). 13.1% (*n* = 66) of participants self‐identified as Hispanic or Latino.

**TABLE 1:** Participant demographics and socioeconomic profile (*N* = 504).

	*n*	%
Sex		
Female	453	89.9
Male	51	10.1
Age (years)		
18–24	7	1.4
25–34	32	6.3
35–44	112	22.2
45–54	144	28.6
55–64	150	29.8
65–74	56	11.1
75+	3	0.6
Race		
White	341	67.7
Black/African American	124	24.6
Asian	24	4.8
2 or more races or other	13	2.8
American Indian or Alaska Native	2	0.2
Ethnicity		
Non‐Hispanic or Latino	438	86.9
Hispanic or Latino	66	13.1
Annual household income		
<$25 000	33	6.5
$25 000–$50 000	74	14.7
$50 000–$75 000	91	18.1
$75 000–$100 000	94	18.7
$100 000–$150 000	97	19.2
≥$150 000	85	16.9
Prefer not to say	30	6.0
Health insurance coverage		
Yes	479	95.0
No	25	5.0
Medication insurance coverage	*n* = 479	
Fully covered	122	25.5
Partially covered	171	35.7
Not covered	178	37.2
Don't know	8	1.7

The mean baseline BMI at the start of the medication was 37.5 ± 8.1 and decreased to 32.07 ± 7.6 at the time of survey. The mean baseline weight prior to initiating GLP‐1 RA therapy was 103.2 kg (±23.6), with a current mean weight of 88.0 kg (±22.5), yielding a mean weight loss of 15.3 kg (±11.7). This represents a mean individual weight loss of 14.6%. A weight loss of ≥ 20 lb/9.1 kg was achieved by 80.4% of participants (*n* = 405). Among the medications and types of formulation reported during this period, compounding pharmacy formulations were most reported (26.8%; *n* = 135), followed by Semaglutide (Ozempic; 26.0%; *n* = 131; Wegovy; 10.5%; *n* = 53), and Tirzepatide (Mounjaro; 22.6%; *n* = 114; Zepbound; 19.6%; *n* = 99). Treatment duration varied widely with 15.1% (*n* = 76) having used the medications for 1 to 3 months, 17.5% (*n* = 88) for 4 to 6 months, 15.9% (*n* = 80) for 7 to 9 months, 13.3% (*n* = 67) for 10 to 12 months, and 38.3% (*n* = 193) for more than 1 year.

Weight loss was the primary driver for drug initiation (70%; *n* = 351), followed by diabetes management (31%; *n* = 156) and overall health improvement (27.5%; *n* = 139). A pre‐existing endocrine or hormonal imbalance diagnosis was reported by 13.9% (*n* = 70) of participants, primarily consisting of thyroid diseases, polycystic ovarian syndrome, perimenopause, and menopause transition. At the time of the survey, 9.3% (*n* = 47) of respondents were receiving concurrent hormone replacement therapy or other hormone‐modulating treatments. Regarding usage of prior weight loss interventions, 17.7% (*n* = 89) of respondents reported previous weight loss drug and procedure interventions, predominantly phentermine (66.2%; *n* = 59), whereas 11.7% (*n* = 59) had opted for weight loss procedures like gastric sleeve (55.7%; *n* = 33) and bariatric surgery (25.4%; *n* = 15).

Overall, 38.7% (*n* = 195) reported cutaneous changes (Table 2). The most reported concerns were facial volume loss (10.9%), followed by skin sagging (8.3%), jowling (7.7%), dryness (6.2%), thinner skin (2.0%), hollowing (1.8%), firmness changes (1.2%), and skin roughness (0.6%). Self‐reported baseline skin type, combination (43.5%) or normal (37.5%), remained stable across treatment durations. Interestingly, perceived skin sensitivity changed, with 8.7% of respondents reporting new sensitive skin perception post‐treatment.

**TABLE 2:** Skin changes reported by GLP‐1 RA users (*N* = 504).

	*n*	%
Skin change		
Any skin change	195	38.7
No changes	309	61.3
Specific changes reported		
Facial volume loss	55	10.91
Sagging	42	8.33
Jowling	39	7.74
Dryness	31	6.20
Thinner skin	10	1.981
Hollowing (sunken appearance)	9	1.79
Firmness changes	6	1.19
Roughness	3	0.60
Timing of onset (*n* = 156 with assessable timing data)		
< 2 months	24	13.46
3–5 months	62	39.74
6–9 months	35	22.44
10–12 months	24	15.38
> 12 months	14	8.97

*Note:* Percentages based on total cohort (*n* = 504) unless otherwise specified.

Abbreviation: GLP‐1 RA, glucagon‐like peptide‐1 receptor agonist.

Timing of onset was assessable in 156 of the 195 cutaneous changes reports (80.0%). 55.1% noticed the cutaneous changes within the first 5 months of initiating the medication: 13.5% (*n* = 21) ≤ 2 months, 39.7% (*n* = 62) between months 3 to 5, 22.4% (*n* = 35) between months 6 to 9, 15.4% (*n* = 24) between months 10 to12, and 9.0% (*n* = 14) after 12 months.

Cutaneous changes correlated strongly with age and cumulative weight loss. The highest prevalence occurred in patients aged ≥ 55 years and those who achieved a weight loss ≥ 20 lb/9.1 kg, with high severity for those losing ≥ 40 lb/18.1 kg. Cutaneous changes were evenly distributed across branded and compounded formulations, suggesting that the cutaneous effects are not driven by a specific pharmacological agent but are likely the result of the weight loss rate, magnitude, and change in metabolic health.

Hair changes were reported by 38.9% (*n* = 196) participants, characterized primarily by increased shedding (20.8%; *n* = 105), thinning (19.0%; *n* = 96), dryness (8.5%; *n* = 43), and breakage (7.9%; *n* = 40), with many respondents indicating multiple concurrent concerns (Table 3). The onset of hair changes occurred significantly earlier than skin changes: 42.0% of respondents reporting shedding within the first 1–3 months of therapy. Hair shedding was particularly pronounced in patients losing ≥ 40 lb/18.1 kg, with 57.0% of them reporting a combination of shedding and thinning. Less frequently reported changes included dullness (4.2%; *n* = 21), brittleness (3.6%; *n* = 18), texture changes (3.6%; *n* = 18), and hair shape changes (2.2%; *n* = 11). 2.8% (*n* = 14) participants reported having a clinical hair disorder diagnosis, with 92.9% of these diagnoses requiring medication initiation.

**TABLE 3:** Hair changes reported by GLP‐1 RA users (*N* = 504).

	*n*	%
Hair changes		
Any hair change	196	38.9
No changes	308	61.1
Specific changes reported^a^		
Increased shedding	105	20.8
Thinning	96	19.0
Dryness	43	8.5
Breakage	40	7.9
Dullness	21	4.2
Brittleness	18	3.6
Texture changes	18	3.6
Hair shape changes (curly ↔ straight)	11	2.2
Hair disorders diagnosed	14	2.8
Diagnosed prior to GLP‐1 RA use	13	92.9
Diagnosed during GLP‐1 RA use	1	7.1
Timing of early onset (1–3 months)^a^		
Shedding		42%
Dryness		17%
Thinning		17%
Breakage		12%

Abbreviation: GLP‐1 RA, glucagon‐like peptide‐1 receptor agonist.

^a^ Multiple responses possible; percentages based on total cohort unless otherwise specified.

We identified statistically significant racial variations in phenotypic hair changes (Table 4). White participants reported higher rates of increased shedding (23.8%) and thinning (22.6%) compared to Black participants, with 9.7% reporting shedding (*p* < 0.001) and 12.9% reporting thinning (*p* < 0.05). Conversely, Black participants reported significantly higher rates of dryness (15.3% vs. 5.9% in White users, *p* < 0.01) and breakage (12.1% vs. 7% in White users, *p* < 0.05).

**TABLE 4:** Hair changes by race in GLP‐1 RA users (*N* = 504).

Hair change category	All respondents *n* (%)	White	Black	*p*‐value White vs. Black/African American
No changes	308 (61.1%)	202 (59.2%)	85 (68.5%)	*p* = 0.06775 (not statistically significant but trending)
Increased shedding	105 (20.8%)	81 (23.8%)	12 (9.7%)	*p* < 0.001 (statistically significant)
Thinning	96 (19%)	77 (22.6%)	16 (12.9%)	*p* < 0.05 (statistically significant)
Dryness	43 (8.5%)	20 (5.9%)	19 (15.3%)	*p* < 0.0001 (statistically significant)
Breakage	40 (7.9%)	24 (7%)	15 (12.1%)	*p* = 0.08181 (not statistically significant but trending)

*Note:* Multiple responses possible: Percentages based on racial subgroup denominators. Only the two largest racial subgroups are presented due to sample size considerations in smaller subgroups.

Abbreviation: GLP‐1RA, glucagon‐line peptide‐1 receptor agonist.

Despite the high rate of negative cutaneous and hair changes, 92.0% of participants reported satisfaction with treatment (58.1% were very satisfied; 33.9% satisfied). Positive quality of life indicators were widespread: 84.0% reported improved body image, 68.5% experienced increased confidence in physical appearance, 76.0% rated their psychological well‐being between 8 to 10, while 59.0% maintained or increased their exercise routines.

## 4 Discussion

This cross‐sectional survey systematically characterizes patient‐reported dermatological outcomes, quality of life, and treatment satisfaction among a cohort of 504 MDWL users. Our findings reveal a distinct clinical paradox: Despite nearly 40% of patients experiencing notable skin (38.7%) and hair (38.9%) changes, overall satisfaction remained high at 92.0%. This satisfaction‐tolerability dissociation indicates that the metabolic, cardiovascular, and psychosocial benefits of medication‐driven weight loss may outweigh aesthetic side effects for some patients. However, these cutaneous and trichological changes remain clinically significant and represent major areas of concern for some patients that warrant their own intervention.

The predominant reported cutaneous manifestations, including facial volume depletion and skin sagging, reflect profound structural changes within the skin. Notably, 55.1% of participants reporting skin changes experienced it rapidly within the first 5 months (21 weeks) of therapy. Among those, over 60% of them already experienced at least 10% of body weight loss. This rapid pace of weight loss is in line with many reported clinical studies [16, 17, 18] and suggests that this speed of MDWL outpaces the skin's natural adaptive capacity for dermal remodeling, leaving redundant tissue that manifests as laxity and jowling. This effect was more pronounced in participants aged > 55 years, a demographic in whom baseline dermal collagen and elastin content are already compromised by menopause [19]. The profound skin laxity observed in this cohort highlights a complex divergence between systemic metabolic improvement and local cutaneous homeostasis. While MDWL via GLP‐1 RAs improves systemic metabolic profiles, including an increase in circulating anti‐inflammatory adiponectin, the local cutaneous microenvironment experiences a rapid catabolic shift. The physical involution of subcutaneous and dermal white adipose tissue (dWAT) removes a critical mechanical scaffold. Furthermore, while systemic adiponectin rises, the depletion of local adipose depots abruptly reduces localized estrogen synthesis and downregulates leptin, both known to stimulate fibroblast proliferation and extracellular matrix synthesis [20]. Consequently, the velocity of adipose deflation outpaces the dermal fibroblasts' adaptive capacity to remodel the collagen network, manifesting clinically as pronounced laxity and jowling [13, 20].

The finding that GLP‐1 RA use does not appear to alter self‐reported baseline skin type (normal, combination, oily) or significantly worsen perceived skin sensitivity is a patient‐reported observation. While reassuring in its consistency across treatment durations, it was not validated against objective dermatological criteria and should therefore be interpreted with caution. Regardless, this perceived skin stability can ensure continued use of patients' established skincare regimens alongside proactive anti‐aging and laxity‐targeted interventions throughout the MDWL journey.

While still emerging, early clinical evidence demonstrates that these targeted approaches can meaningfully address patients' concerns. For instance, a multicenter, open‐label study evaluating a regimen of Poly‐L‐Lactic Acid and Hyaluronic Acid filler injections in 41 participants demonstrated significant clinical improvement in facial definition, contour, and skin radiance as early as week 4 (*p* = 0.0220), with favorable outcomes sustained for up to 9 months [21]. For patients seeking non‐injectable or combination intervention, the literature is equally promising. A randomized, double‐blind, split‐face and split‐neck trial demonstrated that a combination approach—a topical Flavo‐Proxylane regimen (designed to combat glycation and stimulate glycosaminoglycan synthesis) with focused ultrasound—yielded a 16% reduction in skin laxity after 4 weeks of topical monotherapy alone, increasing to a 44% reduction in skin laxity and a 34% reduction in marionette lines by week 12 following a single focused ultrasound session; 94% of participants reported moderate‐to‐significant improvement on the treated side compared with 30% on the placebo side [22]. Further clinical evidence for topical cosmetic formulations demonstrates significant efficacy in preserving skin structural integrity. A 12‐week, open‐label study showed that specialized anti‐aging ingredients resulted in a 20.7% decrease in wrinkle appearance and a 20.1% increase in skin thickness by week 12 (*p* < 0.001), with concomitant improvement in skin firmness [23]. These data underscore the value of integrating proactive topical and procedural strategies into MDWL treatment journeys from the outset of therapy.

### 4.1 Hair Cycle Disruption and Telogen Effluvium

Our findings reveal that hair changes occur significantly earlier than skin changes, with 42% of shedding reported within the 1 to 3 months of weight loss treatment. This rapid onset is consistent with acute telogen effluvium (TE) triggered by the metabolic shock of sudden, severe caloric restriction [7, 15, 24, 25, 26]. Indeed, this temporal onset closely mirrors the kinetics observed following bariatric surgery, where ~57% of patients experience acute telogen effluvium driven by severe catabolic states and rapid micronutrient depletion [27]. This parallel strongly suggests that the MDWL‐induced hair loss is primarily a secondary consequence of rapid diet change rather than a direct toxic effect of the GLP‐1 RAs themselves.

These clinical observations are also strongly corroborated by a recent PRISMA‐compliant systematic review by Branyiczky et al., which identified a consistent association between GLP‐1 RA use and hair loss across a population of over 620, 000 patients, with semaglutide as the strongest pharmacovigilance signal (reporting odds ratio [ROR] 1.24–2.46), followed by tirzepatide (ROR 0.83–1.73) [28]. Branyiczky et al. review also identified cases of hair regrowth associated with GLP‐1 RA use, particularly in patients with improved metabolic parameters, highlighting the bidirectional nature of GLP‐1 RA effects on hair biology, potentially mediated through improved insulin sensitivity, androgen normalization, and enhanced scalp blood flow [28]. The authors proposed four key mechanisms linking GLP‐1 RA therapy to hair loss (1): rapid weight loss triggering TE (2); androgen fluctuations precipitating early‐onset androgenic alopecia (3); GLP‐1 RA–induced appetite suppression leading to nutritional deficiencies; and (4) GLP‐1 RA–related reductions in blood pressure potentially decreasing scalp perfusion [28]. Hair follicles are exquisitely sensitive to deficits in calories, protein, and micronutrients including iron and zinc [25, 29]. GLP‐1 RA–induced appetite suppression may result in inadequate nutrient intake critical for hair follicle maintenance [30, 31]. These findings underscore the need for proactive nutritional monitoring and targeted supplementation strategies in patients undergoing MDWL to correct nutrient deficiencies, which may also reduce TE, but robust clinical evidence is needed.

### 4.2 Racial Variation in Hair Change Phenotypes

A key novelty of this study is the identification of distinct racial patterns in hair change phenotypes. While White users experienced predominantly effluvium‐type changes (shedding, thinning), Black or African American users showed a disproportionate burden of structural hair damage (dryness, breakage). This differential pattern is biologically plausible and clinically meaningful: Black or African American hair possesses a high‐curved elliptical cross‐section and a tightly coiled structure, which results in inherently lower moisture retention and greater susceptibility to mechanical breakage, particularly under conditions of nutritional deficiency and metabolic stress accompanying rapid MDWL [24, 25, 29]. Furthermore, common hair care practices in this population, including chemical processing, heat styling, and high‐tension protective styling, may compound the structural vulnerability. These findings highlight the inadequacy of a universal approach to hair and scalp management in MDWL patients and support the development of personalized recommendations. Moisturizing and strengthening regimens, micronutrient supplementation, scalp stimulation, and low‐level laser therapy may offer benefit for different patients' hair profiles, though clinical evidence is needed to support the recommendations.

### 4.3 Treatment Satisfaction and Quality of Life

Our study reveals a satisfaction‐tolerability paradox: Despite nearly 40% of patients experiencing cutaneous or trichological changes, overall treatment satisfaction remained high at 92.0%. This suggests that the profound psychological and physical improvements associated with weight loss appear to attenuate the negative impact of skin and hair changes on overall self‐perception. However, given the anticipated long‐term nature of GLP‐1 RA therapy [16, 18], the cumulative burden of skin and hair changes may increase over time, potentially eroding this satisfaction equilibrium. Effective clinician‐patient communication can help in balancing expectations and tailoring treatments appropriately.

### 4.4 MDWL Care Recommendations

Last, our findings advocate for a shift in the management of patients undergoing GLP‐1/GIP RA therapy: Transitioning from a reactive, post hoc correction to a preemptive, multimodal care model. MDWL patients may benefit from a proactive combination approach: (1) topicals to fortify the dermal matrix and mitigate laxity; (2) nutritional support to address the early‐onset metabolic shock trigger; and (3) for patients in need of more correction, timely procedures such as energy‐based devices and injectables to address volume loss and structural changes. The goal is to align metabolic success with aesthetic preservation.

### 4.5 Limitations

We acknowledge several limitations associated with this study: First, self‐reported skin and hair changes are subject to recall bias, potential underreporting, and lack objective clinical and biomarker measures. Second, the high prevalence of compound pharmacy formulations (26.8%), which are not FDA‐approved and may vary in purity and dosage, potentially confounds cross‐medication comparisons. Third, the participants were mostly female and therefore the motivations and outcomes may be skewed by gender perspectives; fourth, the usage of previous bariatric procedures may have long‐term physiological implications that may affect the skin and hair; and fifth, other confounding factors beyond GLP1‐RA usage, such as hair styling habits and reproductive hormonal shifts, need further exploration.

## 5 Conclusions

This cross‐sectional survey establishes that dermatological and trichological changes are common, early‐onset, and clinically significant for GLP‐1/GIP RA–mediated weight loss. We demonstrate a correlation between these changes and the magnitude of weight loss, the age of the patients, and distinct racial phenotypes in hair damage patterns, highlighting the necessity for a personalized approach to patient management.

Our findings reveal a notable satisfaction‐to‐tolerability dissociation: While patients remain highly satisfied with the metabolic and well‐being outcomes, the impact on skin and hair presents a significant, often overlooked, unmet clinical need. To bridge this gap, interdisciplinary collaboration between metabolic clinicians and the dermatology community is essential to develop and validate effective and inclusive care solutions, ensuring the pursuit of metabolic health is matched by evidence‐based preservation of patients' skin and hair health.

## Author Contributions

Janet Wangari Olivero: conceptualization, validation, writing. Jyotsna Paturi: conceptualization, project administration, writing. Magali Moreau: conceptualization, data analysis. Kun Qian: analysis. Yaxian Zhen: writing and editing. Stacy‐Ann Stevension: conceptualization, project management. Qian Zheng: conceptualization, project administration. All authors reviewed and approved the final version of the manuscript.

## Ethics Statement

This study was conducted in accordance with Good Clinical Practice (GCP) guidelines and the ethical principles of the Declaration of Helsinki. As a survey‐based observational study involving no more than minimal risk to participants, this study was determined to be exempt from formal institutional review board oversight.

## Consent

Written informed consent was obtained from all participants prior to enrollment.

## Conflicts of Interest

All authors are W2 employees of L'Oréal Research & Innovation USA.

## Data Availability

Research data are not shared.
